# Dynamics of Liver Macrophage Subsets in a Novel Mouse Model of Non-Alcoholic Steatohepatitis Using C57BL/6 Mice

**DOI:** 10.3390/biomedicines11102659

**Published:** 2023-09-28

**Authors:** Nana Makiuchi, Shun Takano, Yuki Tada, Kaichi Kasai, Naoya Igarashi, Koudai Kani, Miyuna Kato, Kana Goto, Yudai Matsuura, Mayuko Ichimura-Shimizu, Yukihiro Furusawa, Koichi Tsuneyama, Yoshinori Nagai

**Affiliations:** 1 Department of Pharmaceutical Engineering, Faculty of Engineering, Toyama Prefectural University, 5180 Kurokawa, Imizu, Toyama 939-0398, Japan; c222731@ed.nagoya-cu.ac.jp (N.M.); u256024@st.pu-toyama.ac.jp (S.T.); yukitada.0101@gmail.com (Y.T.); kinjidog@gmail.com (K.K.); u256001@st.pu-toyama.ac.jp (N.I.); u278001@st.pu-toyama.ac.jp (K.K.); u018002@st.pu-toyama.ac.jp (M.K.); u018006@st.pu-toyama.ac.jp (K.G.); u018028@st.pu-toyama.ac.jp (Y.M.); furusawa@pu-toyama.ac.jp (Y.F.); 2 Department of Pathology and Laboratory Medicine, Tokushima University Graduate School of Biomedical Sciences, 3-8-15 Kuramoto-cho, Tokushima 770-8503, Japan; ichimura.mayuko@tokushima-u.ac.jp (M.I.-S.); tsuneyama.koichi@tokushima-u.ac.jp (K.T.)

**Keywords:** non-alcoholic steatohepatitis, inflammation, fibrosis, macrophage, Kupffer cell, non-alcoholic fatty liver

## Abstract

Macrophages are critical for the development of non-alcoholic steatohepatitis (NASH). Our previous findings in TSNO mouse livers showed that an iHFC (high-fat/cholesterol/cholate) diet induced liver fibrosis similar to human NASH and led to the accumulation of distinct subsets of macrophage: CD11c^+^/Ly6C^−^ and CD11c^−^/Ly6C^+^ cells. CD11c^+^/Ly6C^−^ cells were associated with the promotion of advanced liver fibrosis in NASH. On the other hand, CD11c^−^/Ly6C^+^ cells exhibited an anti-inflammatory effect and were involved in tissue remodeling processes. This study aimed to elucidate whether an iHFC diet with reduced cholic acid (iHFC#2 diet) induces NASH in C57BL/6 mice and examine the macrophage subsets accumulating in the liver. Histological and quantitative real-time PCR analyses revealed that the iHFC#2 diet promoted inflammation and fibrosis indicative of NASH in the livers of C57BL/6 mice. Cell numbers of Kupffer cells decreased and recruited macrophages were accumulated in the livers of iHFC#2 diet-fed C57BL/6 mice. Notably, the iHFC#2 diet resulted in the accumulation of three macrophage subsets in the livers of C57BL/6 mice: CD11c^+^/Ly6C^−^, CD11c^−^/Ly6C^+^, and CD11c^+^/Ly6C^+^ cells. However, CD11c^+^/Ly6C^+^ cells were not distinct populations in the iHFC-fed TSNO mice. Thus, differences in cholic acid content and mouse strain affect the macrophage subsets that accumulate in the liver.

## 1. Introduction

Non-alcoholic fatty liver disease is characterized by fat accumulation in hepatocytes that progresses to hepatic steatosis and is often accompanied by mild inflammation [1]. Some patients with non-alcoholic fatty liver develop non-alcoholic steatohepatitis (NASH), which involves hepatocyte damage, substantial inflammation, and fibrosis [2]. Recently, new nomenclatures of these diseases were announced [3]. Non-alcoholic fatty liver disease and NASH have been replaced by metabolic dysfunction-associated steatotic liver disease and metabolic dysfunction-associated steatohepatitis, respectively [3]. Hepatocytes, hematopoietic cells, and hepatic stellate cells closely interact and contribute to NASH [4]. Recent studies have reported the role of macrophages in promoting NASH development [5]. The liver contains two types of macrophages: Kupffer cells (KCs) and recruited macrophages which are mobilized from the bone marrow to the liver during inflammation [6]. KCs express high F4/80 levels and low CD11b levels (F4/80^Hi^/CD11b^Int^), whereas recruited macrophages exhibit F4/80^Int^ and CD11b^Hi^ phenotypes [6]. Notably, the livers of NASH patients have a unique tissue structure termed the hepatic crown-like structure (hCLS), in which CD11c^+^-recruited macrophages surround dead hepatocytes [7]. The formation of the hCLS is involved in the development of liver fibrosis due to hepatocyte death [8].

Some rodent models of NASH exhibit histological changes resembling human NASH, including stage 3 bridging fibrosis [9]. A high-fat, cholesterol-cholate (iHFC) diet promotes advanced liver fibrosis including stage 3 bridging fibrosis in Tsumura-Suzuki non-obese (TSNO) mice [10]. In a previous study, we examined the phenotypes and dynamics of liver macrophages in iHFC-fed TSNO mice [11]. Flow cytometric analysis revealed two distinct subsets of the recruited macrophages: CD11c^+^/Ly6C^−^ and CD11c^−^/Ly6C^+^ cells [11]. Moreover, our findings indicate that CD11c^+^/Ly6C^−^ cells contribute to the progression of liver fibrosis [11]. On the other hand, CD11c^−^/Ly6C^+^ cells are involved in anti-inflammatory responses [11].

C57BL/6 mice have been frequently used as NASH mouse models in several studies. The administration of carbon tetrachloride to these mice causes hepatic fibrosis followed by hepatocyte necrosis [12]. A methionine- and choline-deficient diet has been frequently used to induce NASH in animals [13]. NASH models also involve using mice deficient in particular genes either with or without the administration of toxic agents [14,15,16], the majority of which are of C57BL/6 mice.

This study aimed to determine whether an iHFC diet induces NASH in C57BL/6 mice and to investigate the differences in NASH pathogenesis in TSNO mice. We used the iHFC#2 diet with reduced cholate content for C57BL/6 mice. Histological and quantitative real-time PCR analyses revealed that the iHFC#2 diet promoted liver inflammation and fibrosis characteristic of NASH in C57BL/6 mice. Similar to iHFC-fed TSNO mice, the iHFC#2-fed C57BL/6 mice showed fewer KCs and more recruited macrophages compared to mice fed a normal diet (ND). Interestingly, the iHFC#2 diet resulted in the accumulation of three macrophage subsets in the livers of C57BL/6 mice: CD11c^+^/Ly6C^−^, CD11c^−^/Ly6C^+^, and CD11c^+^/Ly6C^+^ cells. However, CD11c^+^/Ly6C^+^ cells were not distinct populations in the livers of iHFC-fed TSNO mice. These findings suggest that differences in cholate content and mouse strain affect the macrophage subsets that accumulate in the liver.

## 2. Materials and Methods

### 2.1. Animal Studies

All procedures involving animals were performed according to the ARRIVE reporting guidelines for reporting study design and statistical analysis; experimental procedures; experimental animals and housing and husbandry and the guidelines described in the Proper Conduct of Animal Experiments, as defined by the Science Council of Japan. In addition, the Ethics Committee for Animal Experiment of Toyama Prefectural University approved the animal experiment protocols (No. R1-3 and R4-1).

Male C57BL/6J mice were acquired from Japan SLC (Hamamatsu, Japan) and maintained in SPF conditions at the animal facility of Toyama Prefectural University. Mice had free access to food and water under standard light cycles (12/12 hlight/dark). Ten-week-old male C57BL/6J mice were divided into two groups and fed either an iHFC#2 diet that was high in fat, cholesterol, and cholate (69.8% standard chow, 28.75% palm oil, 1.25% cholesterol, and 0.2% cholate) (Hayashi Kasei, Osaka, Japan) or a normal diet (ND) (Oriental-Yeast, Tokyo, Japan). Mice were anesthetized using isoflurane upon completion of the experiments. Blood and liver were collected for further analysis.

### 2.2. Plasma Chemistry

Plasma concentrations of alanine aminotransferase (ALT), triglyceride (TG), and total cholesterol (T-CHO) were quantified using FUJI DRI-CHEM NX700 analyzer (Fujifilm, Tokyo, Japan), as previously described [11].

### 2.3. Non-Parenchymal Cell Isolation

Non-parenchymal cells were isolated from the livers using the Liver Dissociation Kit (Miltenyi Biotech, Bergisch Gladbach, Germany), as previously described [11].

### 2.4. Flow Cytometry

Non-parenchymal cells (2 × 10^5^) were pretreated with anti-mouse FcγR (2.4G2) antibody for 20 min. Then, the cells were stained with their respective antibodies (Table S1). Dead cells were excluded using 7-amino-actinomycin D (BD Biosciences, San Diego, CA, USA). FACSCantoII (Becton Dickinson & Co., Mountain View, CA, USA) was used for flow cytometry analyses. Data were analyzed using FlowJo software (Version 10.8.1, BD Biosciences).

### 2.5. Quantitative Real-Time PCR

Total RNA was extracted using the NucleoSpin RNA Mini kit (Macherey-Nagel, Düren, Germany). cDNA was obtained via a PrimeScript^®®^ RT reagent kit (Takara Bio Inc., Shiga, Japan). Quantitative real-time PCR was conducted using a FastStart Universal Probe Master (Roche Applied Science, Mannheim, Germany), as previously described [11]. TaqMan probes used are provided in Table S2 (Applied Biosystems, Carlsbad, CA, USA).

### 2.6. Liver Histology and Immunohistochemistry

Hematoxylin and eosin, Sirius red, and immunohistochemical staining were performed as previously described [11]. Positive areas for Sirius red, F4/80, CD11c, and Ly6C were quantified using ImageJ software, Version 1.53t. Histological scores and grades were assessed according to the previous literature [17]. All histological analyses were performed in a blinded manner.

### 2.7. Statistical Analysis

Statistical differences were analyzed using Student’s *t*-test or Welch’s *t*-test for the two groups’ comparison by GraphPad Prism 9 software (GraphPad; San Diego, CA, USA). *p* < 0.05 was considered statistically significant. Statistical difference was determined as follows: *** *p* < 0.001, ** *p* < 0.01, * *p* < 0.05. Statistical results are expressed as means ± standard deviation (SD).

## 3. Results

### 3.1. iHFC#2 Diet Induces Inflammation, Steatosis, Hepatocyte Ballooning, and Fibrosis in the Livers of C57BL/6 Mice

**Figure 1:**
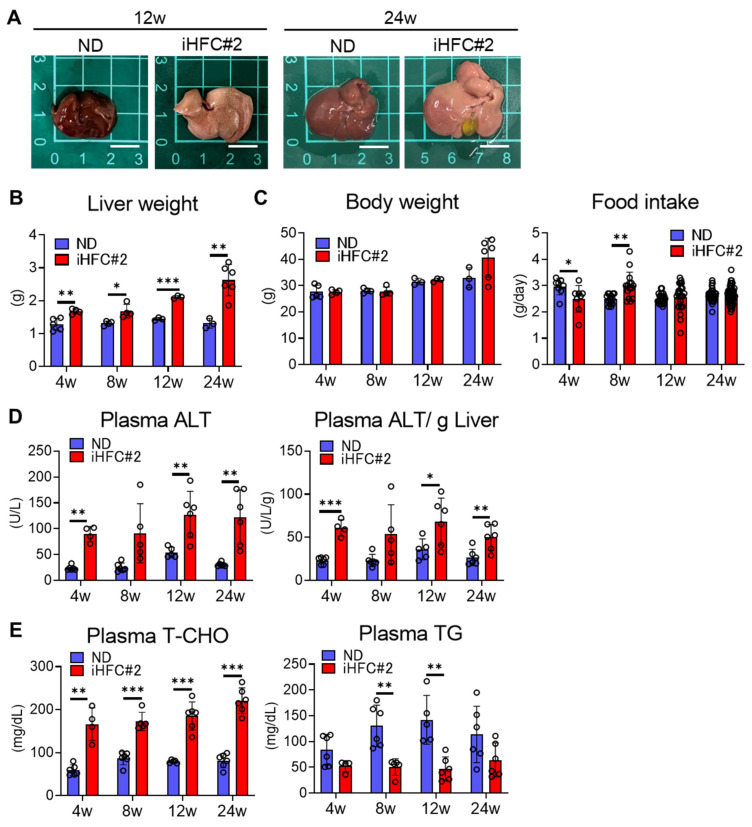
iHFC#2 diet induces increases in liver weight and plasma ALT levels. (**A**) Representative photos of the livers from C57BL/6 mice fed with the normal diet (ND) or iHFC#2 diet for the indicated time periods. Scale bars, 1 cm. (**B**) Liver weights of C57BL/6 mice (n = 3 to 6). (**C**) Body weights and daily food intakes were measured for C57BL/6 mice fed with the ND or iHFC#2 diet for the indicated time periods (n = 6 to 18). (**D**) Left, plasma ALT levels of C57BL/6 mice (n = 4 to 6). Right, plasma ALT levels were normalized to liver weight (in grams) for calculation (n = 4 to 6). (**E**) Plasma T-CHO and TG levels were measured for C57BL/6 mice fed with the ND or iHFC#2 diet for the indicated time periods (n = 4 to 6). Data are shown as means ± SD. * *p* < 0.05, ** *p* < 0.01, *** *p* < 0.001.

We investigated whether iHFC#2-fed C57BL/6 mice exhibited NASH-related pathological changes. In preliminary experiments, an iHFC diet containing 0.5% cholate was found to cause lethal liver injury in C57BL/6 mice. Therefore, we used a 0.2% cholate iHFC#2 diet in this study. iHFC#2-fed mice had larger and paler livers than ND-fed mice (Figure 1A), and their liver masses significantly increased compared with those from ND-fed mice (Figure 1B). No significant difference was observed in the body mass between mice consuming the ND and iHFC#2 diets (Figure 1C, left). Significant differences in average daily food intake were observed at 4 and 8 weeks between the ND and iHFC#2 diets (Figure 1C, right). Plasma alanine aminotransferase (ALT) activity increased in mice following 4 weeks of iHFC#2 diet feeding (Figure 1D). iHFC#2-fed mice exhibited consistently high plasma total cholesterol (T-CHO) concentrations (Figure 1E, left) and lower plasma triglyceride (TG) concentrations compared to ND-fed mice (Figure 1E, right).

**Figure 2:**
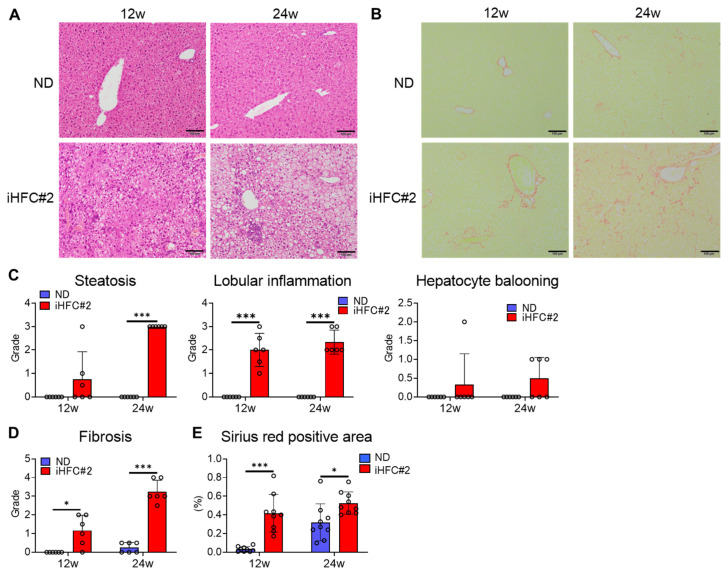
iHFC#2 diet induces steatohepatitis and fibrosis. (**A**) Hematoxylin and eosin-stained liver sections. Scale bars, 100 μm. (**B**) Sirius red-stained sections of the livers. Scale bars, 100 μm. (**C**) Steatosis (0 to 3), lobular inflammation (0 to 3), and hepatocyte ballooning (0 to 2) were evaluated (n = 6). (**D**) Liver fibrosis (0 to 4) was evaluated (n = 6). (**E**) Three locations were captured per three liver sections for each group. Subsequently, the positive areas for Sirius red were quantified at nine locations using ImageJ software, Version 1.53t. Data are shown as means ± SD. * *p* < 0.05, *** *p* < 0.001.

Mild steatosis developed after 12 weeks of the iHFC#2 diet and worsened with a longer feeding duration (Figure 2A,C). In addition, lobular inflammation was observed at weeks 12 and 24 (Figure 2A,C). Mild hepatocyte ballooning was observed in the iHFC#2 group at both time points (Figure 2A,C). Furthermore, perivenular and perisinusoidal fibrosis similar to human NASH was observed after 12 weeks of the iHFC#2 diet (Figure 2B,D). The fibrosis exhibited a progressive expansion, with the emergence of bridging fibrosis becoming evident after 24 weeks on the iHFC#2 diet (Figure 2B,D). The areas positive for Sirius red staining were significantly larger compared to mice fed an ND after 12 weeks of iHFC#2 diet feeding (Figure 2E). These data demonstrate that the iHFC#2 diet induces inflammatory and fibrotic changes characteristic of NASH in the livers of C57BL6 mice.

### 3.2. iHFC#2 Diet Increases the mRNA Expression of Inflammation- and Fibrosis-Related Genes in the Livers of C57BL/6 Mice

**Figure 3:**
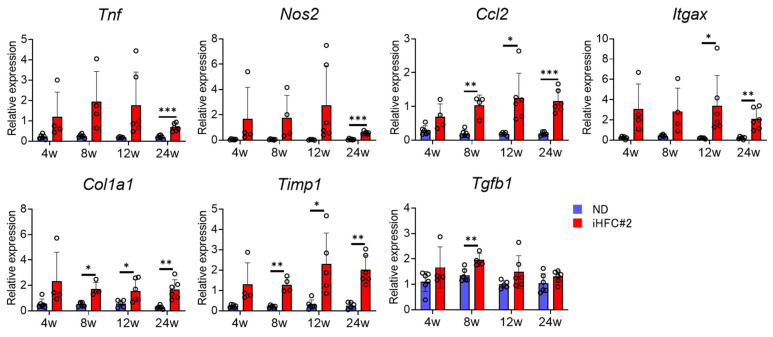
iHFC#2 diet elevates the expression levels of genes associated with inflammation and fibrosis in the liver. RT-qPCR of TNF-α, iNOS, CCL2, CD11c, collagen type 1, TIMP-1, and TGF-β mRNA in the livers (n = 4 to 6). Data are shown as means ± SD. * *p* < 0.05, ** *p* < 0.01, *** *p* < 0.001.

Next, we measured the expression levels of inflammatory and fibrotic genes in the liver of ND- or iHFC#2-fed mice. The expression levels of TNF-α (*Tnf*), CCL2 (*Ccl2*), and M1 macrophage markers iNOS and CD11c (*Nos2* and *Itgax*) exhibited a substantial increase during the early stages of the iHFC#2 diet (Figure 3). Consistent with the histological data (Figure 2B,D,E), the expression levels of collagen 1 (*Col1a1*) mRNA were higher in the livers of iHFC#2-fed mice than in those of ND-fed mice (Figure 3). iHFC#2 feeding led to a significant upregulation in the expression levels of Timp-1 (*Timp1*) mRNA, which plays a role in regulating extracellular matrix degradation (Figure 3). There was a significant difference observed in the expression levels of TGF-β (*Tgfb1*) mRNA, which is associated with extracellular matrix production, between the ND and iHFC#2 diet groups at 8 weeks (Figure 3). Thus, the iHFC#2 diet increased the expression levels of inflammation- and fibrosis-related genes in the livers of C57BL/6 mice.

### 3.3. iHFC#2 Diet Induces the Infiltration of F4/80^Int^/CD11b^Int-Hi^-Recruited Macrophages in the Livers of C57BL/6 Mice

**Figure 4:**
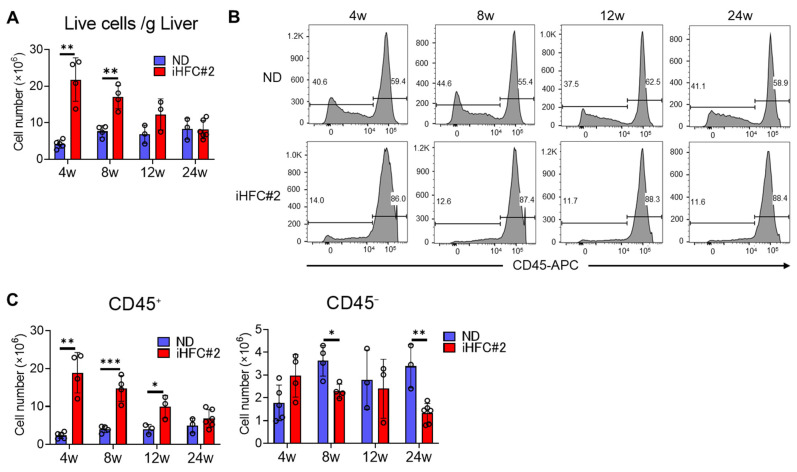
iHFC#2 diet induces the accumulation of CD45^+^ leukocytes in the livers of C57BL/6 mice. (**A**) Cell number of live non-parenchymal cells in the livers from C57BL/6 mice fed with the ND or iHFC#2 diet for the indicated time periods (n = 3 to 6). (**B**) Representative flow cytometry data of CD45 expression in live non-parenchymal cells of the livers from C57BL/6 mice fed with the ND or iHFC#2 diet for the indicated time periods. (**C**) Cell number of CD45^+^ or CD45^−^ live non-parenchymal cells was determined by flow cytometry analysis done in (n = 3 to 6). Data are shown as means ± SD. * *p* < 0.05, ** *p* < 0.01, *** *p* < 0.001.

The number of non-parenchymal cells exhibited a significant increase at 4 weeks of iHFC#2 diet, followed by a gradual decline over time (Figure 4A). The iHFC#2 diet-fed mice showed a higher proportion of CD45^+^ leukocytes, regardless of the feeding duration (Figure 4B). The number of CD45^+^ cells reached its peak after 4 weeks of iHFC#2 diet feeding, and then, gradually declined (Figure 4C, left). Significant differences in the number of CD45^−^ cells were observed at 8 and 24 weeks between the ND and iHFC#2 diets (Figure 4C, right).

**Figure 5:**
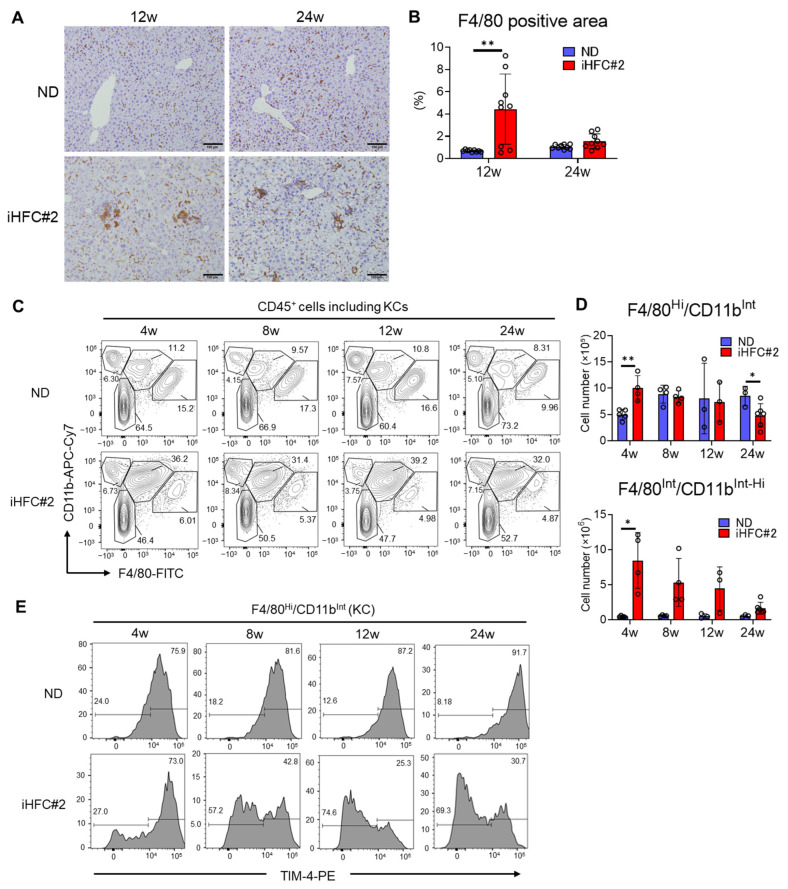
iHFC#2 diet leads to the accumulation of F4/80^Int^/CD11b^Int-Hi^-recruited macrophages in the livers of C57BL/6 mice. (**A**) Histological images of F4/80 immunostaining. Scale bars, 100 μm. (**B**) Three locations were captured per three liver sections for each group. Subsequently, the positive areas for F4/80 were quantified at nine locations using ImageJ software, Version 1.53t. (**C**) CD11b and F4/80 expression in CD45^+^ non-parenchymal cells. (**D**) Cell number of F4/80^Hi^/CD11b^Int^ KCs (upper) and F4/80^Int^/CD11b^Int-Hi^-recruited macrophages (lower) were calculated (n = 3 to 6). (**E**) Representative flow cytometry data of TIM-4 expression in F4/80^Hi^/CD11b^Int^ KCs of the livers from C57BL/6 mice on the ND or iHFC#2 diet for the indicated time periods (n = 3 to 6). Data are shown as means ± SD. * *p* < 0.05, ** *p* < 0.01.

To investigate the roles of macrophages in the pathogenesis of iHFC#2 diet-induced NASH, we examined the expression of the pan-macrophage marker F4/80 in the livers of C57BL/6 mice. Immunohistochemical staining revealed a significant increase in the F4/80^+^ area after 12 weeks of iHFC#2 feeding (Figure 5A,B). As KCs have high autofluorescence [11] (Figure S1), two different gating strategies were used to examine the CD45^+^ cells, depending on whether KCs were included in the analysis (Figure S2) [11]. Similar to iHFC diet-fed TSNO mice [11], C57BL/6 mice fed an ND also exhibited populations expressing F4/80 and/or CD11b in their livers (Figure 5C). These included F4/80^−^/CD11b^Hi^ neutrophils, F4/80^Int^/CD11b^Int-Hi^-recruited macrophages, and F4/80^Hi^/CD11b^Int^ KCs (Figure 5C). The percentages of F4/80^−^/CD11b^Hi^ neutrophils showed no significant differences between the ND- and iHFC#2-fed mice (Figure 5C). Significant differences in the number of F4/80^Hi^/CD11b^Int^ KCs were observed at 4 and 24 weeks between the ND and iHFC#2 groups (Figure 5D, upper). The KC-specific marker TIM-4 was highly expressed in F4/80^Hi^/CD11b^Int^ KCs under normal conditions (Figure 5E) [18]. Consistent with previous findings [11,19], the proportion of KCs lacking TIM-4 expression showed a gradual increase as the duration of iHFC#2-feeding extended (Figure 5E). Of interest, the iHFC#2 diet markedly increased the number of F4/80^Int^/CD11b^Int-Hi^-recruited macrophages, with a peak at week 4 (Figure 5D, lower).

### 3.4. Recruited Macrophages Include Three Subsets Characterized by Distinct Markers: CD11c^−^/Ly6C^+^, CD11c^+^/Ly6C^−^, and CD11c^+^/Ly6C^+^ Cells

**Figure 6:**
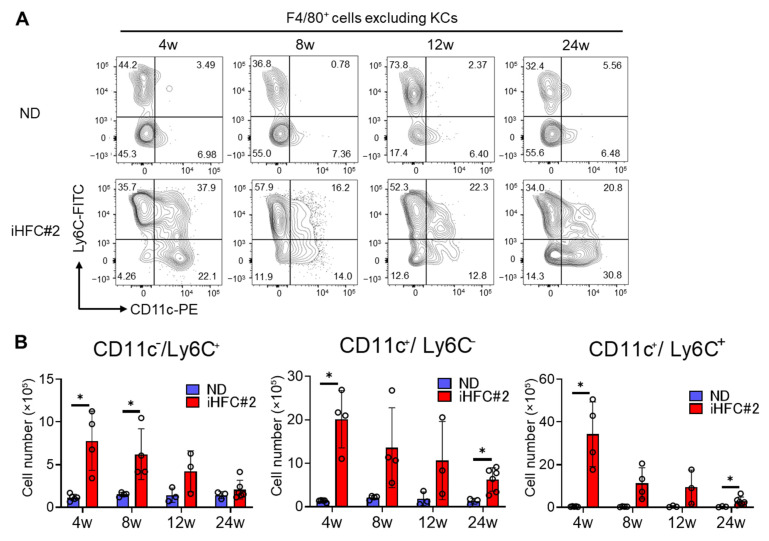
iHFC#2 diet leads to the accumulation of three types of recruited macrophage subsets in the liver of C57BL/6 mice. (**A**) The expression of CD11c and Ly6C was analyzed in F4/80^+^-recruited macrophages. (**B**) Cell number of CD11c^+^/Ly6C^−^, CD11c^−^/Ly6C^+^, and CD11c^+^/Ly6C^+^ cells were calculated (n = 3 to 6). Data are shown as means ± SD. * *p* < 0.05.

To identify the cell populations of F4/80^Int^/CD11b^Int-Hi^-recruited macrophages, we examined F4/80^+^ live single cells, excluding KCs (Figure S3A,B), which mostly consist of recruited macrophages. The F4/80^+^ macrophages, which excluded KCs, included three subsets: CD11c^−^/Ly6C^+^, CD11c^+^/Ly6C^−^, and CD11c^+^/Ly6C^+^ cells, in the livers of iHFC#2-fed mice (Figure 6A). In contrast to TSNO mice [11], C57BL/6 mice exhibited a higher percentage of CD11c^−^/Ly6C^+^ cells during ND feeding (Figure 6A). The number of this subset increased significantly with iHFC#2 diet feeding at week 4 and 8 but gradually decreased thereafter (Figure 6B, left). Additionally, the number of CD11c^+^/Ly6C^−^ peaked at week 4 of the iHFC#2 diet, reaching approximately three times the number of CD11c^−^/Ly6C^+^ subsets (Figure 6B, middle). Interestingly, iHFC#2-fed C57BL/6 mice showed an increased percentage of cells expressing both CD11c and Ly6C (Figure 6A), which was not distinct in iHFC-fed TSNO mice [11]. Furthermore, the number of this subset peaked at week 4 of the iHFC#2 diet and was the highest among the three subsets (Figure 6B, right). 

**Figure 7:**
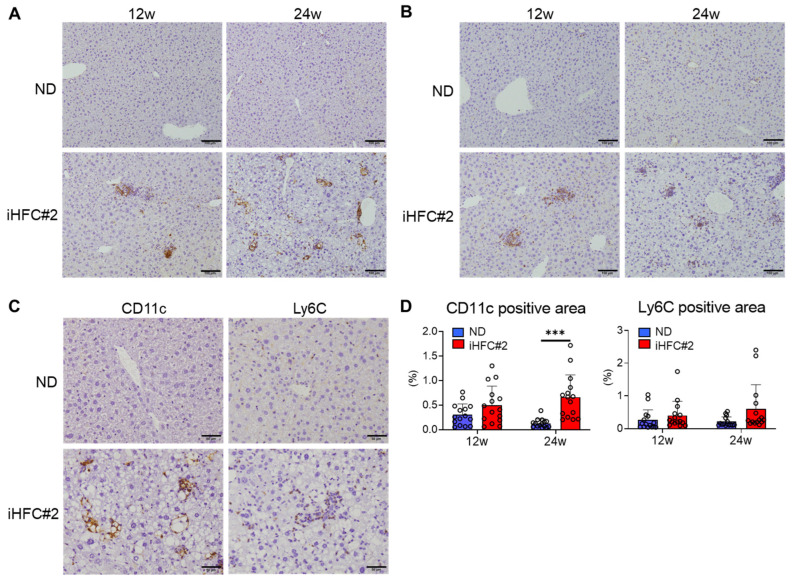
iHFC#2 diet induces the accumulation of CD11c^+^ and Ly6C^+^ cells in the liver of C57BL/6 mice. (**A**,**B**) Representative histological images of CD11c (**A**) and Ly6C (**B**) immunostaining of the livers from C57BL/6 mice on the ND or iHFC#2 diet for the indicated time periods. Scale bars, 100 μm. (**C**) Representative histological images of CD11c and Ly6C immunostaining of the livers from C57BL/6 mice on the ND or iHFC#2 diet. Scale bars, 50 μm. (**D**) Five locations were captured per three liver sections for each group. Subsequently, the positive areas for CD11c or Ly6C were quantified at fifteen locations using ImageJ software, Version 1.53t. Data are shown as means ± SD. *** *p* < 0.001.

CD11c-positive cell accumulation was observed after 12 weeks of iHFC#2 diet feeding and significantly increased after 24 weeks (Figure 7A,D, left). Magnified images of 24 weeks of iHFC#2-diet feeding revealed CD11c-positive cells forming hCLSs around the lipid droplets (Figure 7C). Ly6C-positive cells accumulated after 12 weeks of iHFC#2 feeding (Figure 7B). However, no significant difference in the percentage of Ly6C-positive cells was observed between the ND and iHFC#2 groups (Figure 7D, right). Unlike CD11c-positive cells, Ly6C-positive cells did not form hCLSs (Figure 7C). These results suggest that F4/80^+^-recruited macrophages consist of three different subsets, CD11c^−^/Ly6C^+^, CD11c^+^/Ly6C^−^, and CD11c^+^/Ly6C^+^ cells, in the livers of iHFC#2-fed C57BL/6 mice and CD11c^+^ and Ly6C^+^ cells are localized to different locations in the livers.

## 4. Discussion

In this study, we analyzed NASH development in C57BL/6 mice fed a reduced cholate iHFC#2 diet. After 24 weeks on the iHFC#2 diet, C57BL/6 mice developed stage 3 liver fibrosis similar to iHFC-fed TSNO mice. Several diet-induced NASH models, such as a methionine- and choline-deficient diet- or choline-deficient, L-amino acid-defined diet-fed NASH model mice, exhibit body weight loss [20]. On the other hand, the iHFC#2-fed C57BL/6 mouse model showed a gradual body weight gain (Figure 1C), indicating that this model is considered an obese NASH model. Interestingly, C57BL6 mice had more Ly6C^+^/CD11c^−^ macrophages in their livers compared to TSNO mice on ND. Furthermore, the iHFC#2 diet increased the number of CD11c^+^/Ly6C^+^ macrophages in C57BL/6 mice but not in TSNO mice. These results demonstrate that different strains of mice have different macrophage subsets in the liver at a steady state. Furthermore, the cholate content of the iHFC diet and mouse strains may affect macrophage dynamics in the liver.

iHFC#2-fed C57BL/6 mice showed perivenular and perisinusoidal fibrosis as in iHFC-fed TSNO mice (Figure 2B,D) [11]. C57BL/6 mice fed iHFC#2 for 24 weeks also showed bridging fibrosis (Figure 2B). These results indicate that iHFC#2 feeding with a reduced cholate content develops advanced liver fibrosis similar to human NASH. On the other hand, iHFC#2-fed C57BL/6 mice showed less liver damage, lobular inflammation, and hepatocyte ballooning than iHFC-fed TSNO mice (Figure 1D and Figure 2C) [11]. Thus, differences in cholate content and mouse strain may influence these pathological changes of NASH.

In our previous study, an iHFC-diet feeding resulted in the accumulation of CD11c^+^/Ly6C^−^ and CD11c^−^/Ly6C^+^ macrophages in the livers of TSNO mice [11]. CD11c^+^/Ly6C^−^ macrophages accumulated in the livers of iHFC#2-fed C57BL/6 mice and formed hCLSs, similar to those in iHFC-fed TSNO mice (Figure 6A and Figure 7C) [11]. This indicates that CD11c^+^/Ly6C^−^ macrophages play an important role in liver fibrosis, regardless of the mouse strain or diet. In contrast, CD11c^−^/Ly6C^+^ macrophages in TSNO mice accumulated in the livers after the iHFC diet, whereas a significant proportion of this subset was resident in the livers of ND-fed C57BL/6 mice (Figure 6A). Several studies have reported the role and dynamics of Ly6C^+^ macrophages in NASH pathogenesis [21,22,23,24]. Ly6C^+^ macrophages play anti-inflammatory roles in the liver by producing mediators such as IL-10 and arginase-1 [22,23], which is consistent with our previous results [11]. Thus, the milder liver damage, lobular inflammation, and hepatocyte ballooning associated with NASH of C57BL/6 mice compared to TSNO mice may be attributed to the higher number of CD11c^−^/Ly6C^+^ macrophages at steady state.

CD11c^+^ macrophages accumulate in the liver of not only obese but also lean NASH models. A methionine- and choline-deficient diet-induced model is a commonly used NASH animal model, but it causes weight loss early in the feeding period [20]. It has been reported that CD11c-positive monocyte-derived macrophages accumulate in the liver after the diet feeding [25]. Furthermore, it has been suggested that anti-inflammatory macrophages also accumulate in the liver of this model [26], but it is unclear whether these express Ly6C. The CD11c^−^/Ly6C^+^ macrophage may be a unique cell population of the iHFC- and iHFC#2-diet-induced NASH model.

Interestingly, CD11c^+^/Ly6C^+^ macrophages were found in iHFC#2 diet-fed C57BL/6 mice (Figure 6A). The number of this subset, along with CD11c^+^/Ly6C^−^ and CD11c^−^/Ly6c^+^ macrophages, peaked after 4 weeks of feeding and declined thereafter (Figure 6B). Therefore, CD11c^+^/Ly6C^+^ macrophages may be the precursors or transitional cells of the other two macrophage subsets. Future studies should investigate their gene expression profiles to determine whether CD11c^+^/Ly6C^+^ cells exhibit an intermediate phenotype between CD11c^+^/Ly6C^−^ and CD11c^−^/Ly6C^+^ cells.

Changes in gut microbiota and bile acid composition also affect the progression of chronic liver diseases [27,28,29]. We previously reported that changes in liver inflammation and fibrosis induced by the iHFC diet in TSNO mice can be affected by modifications in the gut microbiota and bile acid composition [30]. Further investigations are necessary to explore the impact of these mechanisms on the development of NASH pathogenesis in C57BL/6 mice fed the iHFC#2 diet.

In conclusion, our results provided valuable insights into the distinct subsets of liver macrophage subsets using a NASH C57BL/6 mouse model. Our findings may provide information that will aid in the development of therapeutic agents for NASH that target the macrophage subsets. Since most gene-deficient mice are from a C57BL/6 background, the iHFC#2-fed NASH model will be useful in analyzing the function of various genes in obese NASH. This information will lead to a better understanding of NASH pathogenesis and the development of therapeutic interventions.

## Data Availability

The data that support the findings of this study are available from the corresponding author upon reasonable request.

## Supplementary Materials

The following supporting information can be downloaded at: https://www.mdpi.com/article/10.3390/biomedicines11102659/s1, Figure S1: Representative flow cytometry data of CD45, TIM-4, F4/80, and CD11b expressions on live non-parenchymal cells of the livers from C57BL/6 mice; Figure S2: Gating strategy for flow cytometry analysis of non-parenchymal cell of the liver from C57BL/6 mice; Figure S3: Gating strategy for flow cytometry analysis of F4/80+ non-parenchymal cell, excluding KCs, of the liver from C57BL/6 mice; Table S1: Antibodies for flow cytometry; Table S2: Primers for RT-qPCR.

## Abbreviations

ALT	alanine aminotransferase
hCLS	hepatic crown-like structure
KC	Kupffer cell
NASH	non-alcoholic steatohepatitis
ND	normal diet
T-CHO	total cholesterol
TG	triglyceride
TSNO	Tsumura-Suzuki non-obese
