# Supplementary material for: Dynamics of Liver Macrophage Subsets in a Novel Mouse Model of Non-Alcoholic Steatohepatitis Using C57BL/6 Mice
Source: Biomedicines. 2023 Sep 28;11(10):2659. doi: 10.3390/biomedicines11102659 (PMC10604124; doi:10.3390/biomedicines11102659)
Supplement: Supplementary file 1 [file biomedicines-11-02659-s001.zip › biomedicines-2615779-supplementary.pdf]

Antibody	Clone	Conjugate	Source
Anti-CD11c	HL3	PE	BD Pharmingen
Anti-CD11b/Mac-1	M1/70	APC-Cy7	BD Pharmingen
Anti-CD45	30-F11	APC	BioLegend
Anti-F4/80	BM8	PE	BioLegend
Anti-F4/80	BM8	FITC	BioLegend
Anti-TIM-4	RMT4-54	PE	BioLegend
Anti-Ly6C	HK1.4	FITC	BioLegend

Supplementary Table S1. Antibodies for flow cytometry.

The antibodies for flow cytometry are listed. The antibodies were purchased from BD Pharmingen (San Diego, CA) or BioLegend (San Diego, CA).

Gene	Gene Symbol	Gene Name	Assay ID
Hprt	Hprt1	hypoxanthine guanine phosphoribosyl transferase	Mm00446968_m1
TNF-a	Tnf	tumor necrosis factor	Mm00443258_m1
iNOS	Nos2	nitric oxide synthase 2, inducible	Mm01309898_m1
MCP-1	Ccl2	chemokine (C-C motif) ligand 2	Mm00441243_g1
CD11c	Itgax	integrin alpha X	Mm00498698_m1
Col1a-1	Col1a1	collagen, type 1, alpha 1	Mm00801666_g1
TIMP-1	Timp1	tissue inhibitor of metalloproteinase 1	Mm00441818_m1
Tgfb-1	Tgfb1	transforming growth factor, beta 1	Mm01178820_m1

Supplementary Table S2. Primers for RT-qPCR.

The primers were purchased from Applied Biosystems (Waltham, MA).

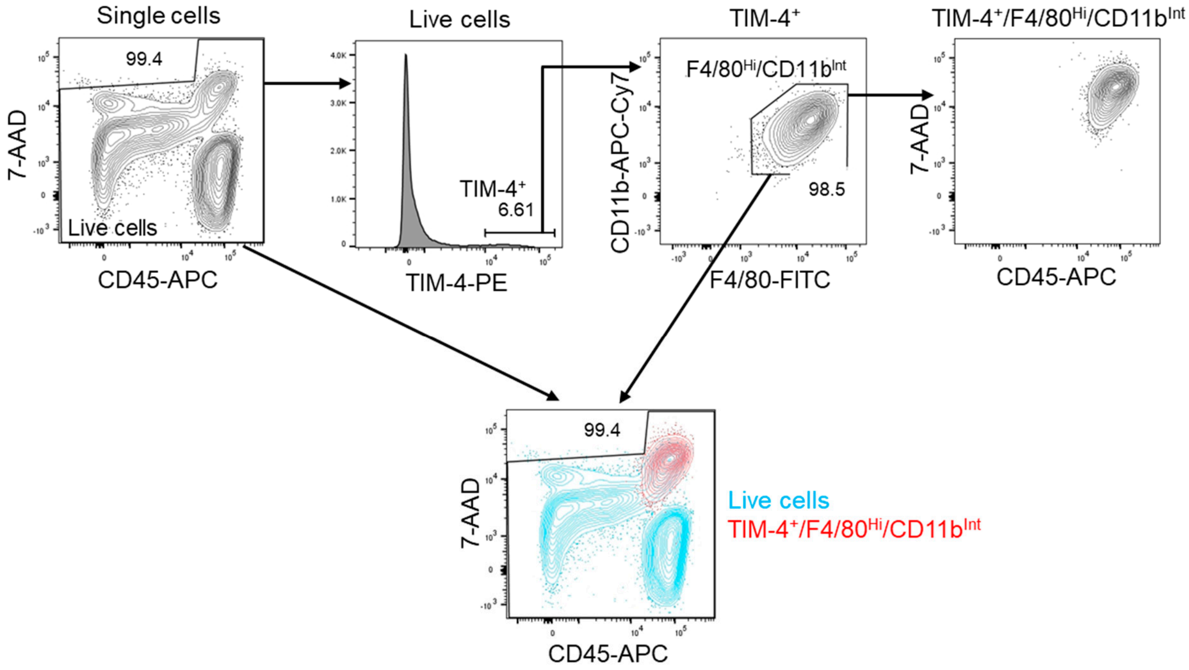

Supplementary Figure S1. Representative flow cytometry data of CD45, TIM-4, F4/80, and CD11b expressions on live non-parenchymal cells of the livers from C57BL/6 mice. Single non-parenchymal cells of the livers from ND-fed C57BL/6 mice contained highly auto-fluorescent $CD45^+$ cells. Staining of these cells with an antibody to TIM-4, a specific marker of KCs, revealed that TIM-4^+ KCs were $F4/80^{Hi}/CD11b^{Int}$ and highly auto-fluorescent $CD45^+$ cells.

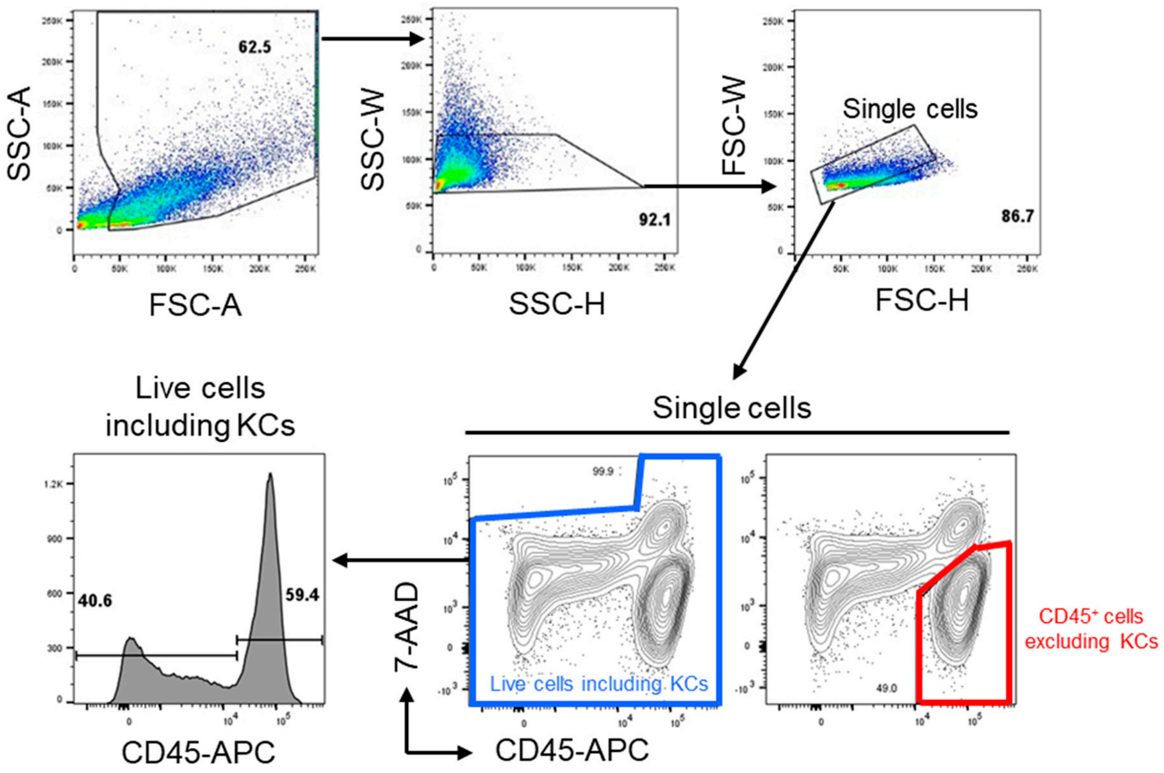

Supplementary Figure S2. Gating strategy for flow cytometry analysis of non-parenchymal cell of the liver from C57BL/6 mice.

Since KCs were highly auto-fluorescent $CD45^+$ cells (Figure S1), we used two different gating strategies to analyze $CD45^+$ cells, depending on whether the KCs were being assessed or not. To examine $CD45^+$ cells including KCs, single cells were first analyzed with a plot of CD45 and 7-AAD and gated on live cells including highly fluorescent $CD45^+$ cells (blue gate), followed by a histogram of CD45. To examine $CD45^+$ cells excluding KCs, single cells were gated on CD45 expressing cells excluding highly fluorescent $CD45^+$ cells (red gate).

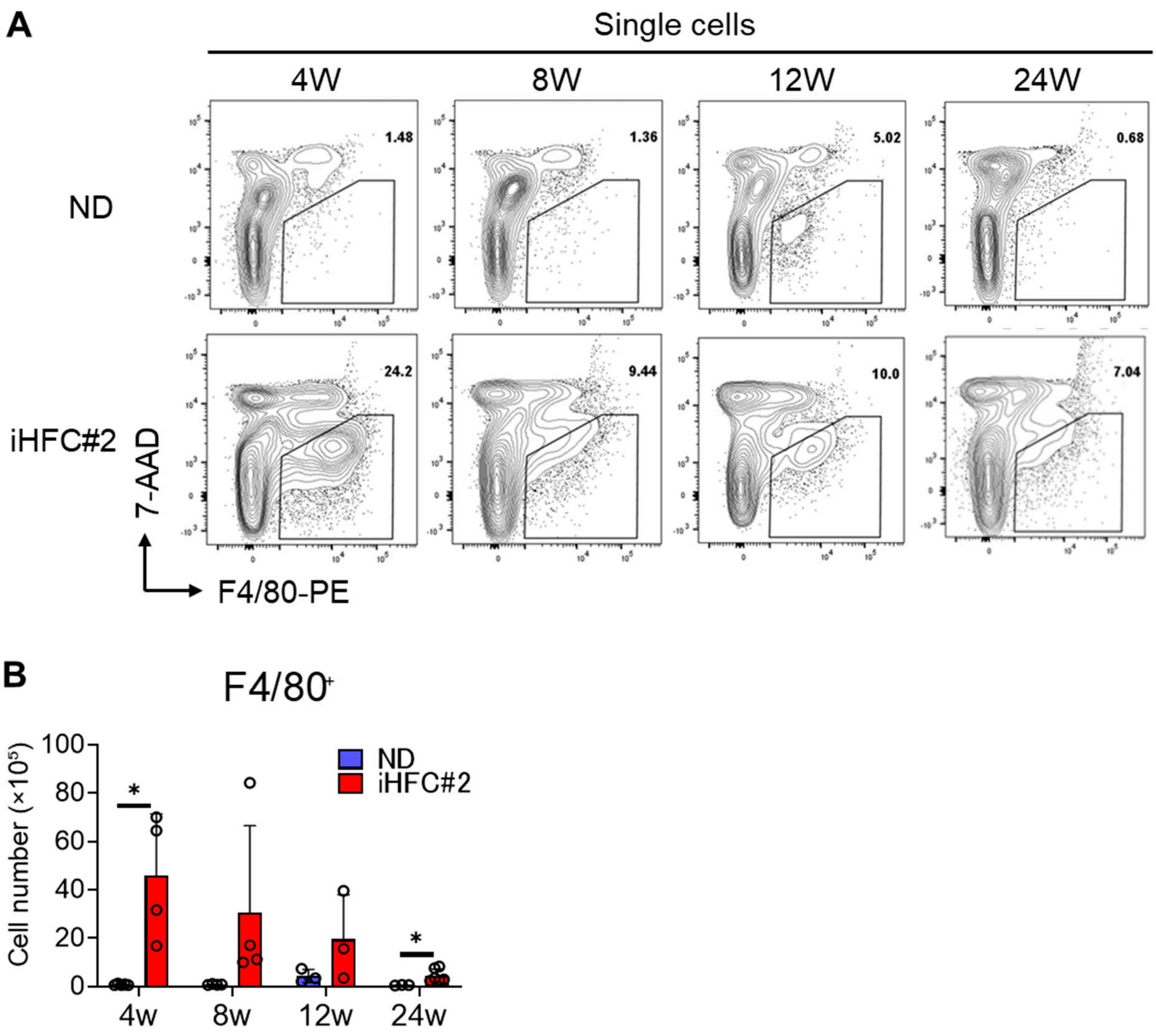

Supplementary Figure S3. Gating strategy for flow cytometry analysis of F4/80$^+$ non-parenchymal cell, excluding KCs, of the liver from C57BL/6 mice.

(A) Non-parenchymal cells were isolated from the liver from ND- or iHFC#2 diet-fed C57BL/6 mice for the indicated weeks. Single non-parenchymal cells were analyzed with a plot of F4/80 and 7-AAD and gated on live F4/80$^+$ cells excluding dead cells and highly fluorescent F4/80-positive KCs. Then, we examined the expression of Ly6C and CD11c on F4/80$^+$ recruited macrophages, as shown in Figure 6A. (B) The cell number of F4/80$^+$ recruited macrophages was determined by flow cytometry analysis done in Supplementary Fig. 3A (n = 3 or 6 per group). Data are shown as means ± SD. *p <0.05
